# Carrot-based fermentation juice rich in sleep-promoting components improved sleep in mice

**DOI:** 10.3389/fnut.2022.1043055

**Published:** 2022-11-29

**Authors:** Daiyao Liu, Jianming Zhang, Juan Chen, Chengcheng Zhang, Huaxi Yi, Daqun Liu

**Affiliations:** ^1^College of Food Science and Engineering, Ocean University of China, Qingdao, Shandong, China; ^2^Institute of Food Science, Zhejiang Academy of Agricultural Sciences, Hangzhou, Zhejiang, China

**Keywords:** *Levilactobacillus brevis* YSJ3, fermented carrot juice, sleep-promoting components, improved sleep, mice

## Abstract

The impact of fermentation by *Levilactobacillus brevis* YSJ3 on sleep-promoting components (SPCs) of carrot juice was evaluated. The contents of acetic acid, isovaleric acid, butyric acid, and γ-aminobutyric acid (GABA) significantly increased after fermentation. The beneficial effects of fermented carrot juice (FCJ) on sleep were evaluated in animal experiments. Behavioral test reveal SPCs-enriched FCJ could effectively relieve anxiety. The sleep duration in the FCJ group were extended compared to the control (NC) group and the unfermented carrot juice (UCJ) group. Moreover, the relative abundances of *Ruminiclostridium* and *Akkermansia* in the FCJ group and PC group, respectively, increased significantly, compared to the NC group the UCJ group. The contents of gut short-chain fatty acids in the FCJ group were significantly higher than that in the NC group and the UCJ group. The levels of GABA and 5-hydroxytryptamine in the brain for the FCJ group also increased significantly, compared to the NC group and the UCJ group. It indicated that SPCs-enriched FCJ effectively improved sleep in mice, which might be related to the fermentation of carrot juice and the compounds produced during the fermentation.

## Introduction

Insufficient sleep is increasingly problematic in modern society. Sufficient sleep is important for maintaining physical and mental health; yet, nearly a third of adults get less than 6 h of sleep a day (1). Insufficient sleep has been associated with a variety of adverse medical problems, such as obesity, diabetes, cardiovascular disease, neurocognitive impairment, and weakened immunity (2–5). Benzodiazepines and non-benzodiazepines are widely used to address insufficient sleep (6). However, these drugs are not without serious side effects, such as drug dependence and withdrawal reactions (7). As a result, diet modifications and dietary supplements have become alternate approaches to complementary and alternative medicine (8).

According to a recent report, many foods (animal-based foods, fruits, and vegetables) contain sleep-promoting components (SPCs), such as neurotransmitters (8) and short-chain fatty acids (SCFAs) (9). In particular, γ-aminobutyric acid (GABA), 5-hydroxytryptamine (5-HT), and butyrate have been linked to sleep (10–12). Indeed, dietary supplements of these components can effectively improve sleep quality (8). In addition, probiotics are also considered to be SPCs. Lin et al. found that *Limosilactobacillus fermentum* PS150™ could be used as a potential dietary supplement to improve sleep in pentobarbital-induced mice (13). Lactic fermentation is widely used in food processing, and lactic fermentation can enrich many active substances in foods, such as glucosinolates, peptides, SCFAs, neurotransmitters, vitamins, and exopolysaccharides (14). For example, Fuente et al. used *Levilactobacillus brevis* POM and *Lactiplantibacillus plantarum* (TR-7, TR-71, TR-14) to assist in the fermentation of an orange beverage, with high antioxidant activity (15). Wei et al. used *Lactiplantibacillus plantarum* B7 and C8-1 to ferment bog bilberry juice, and the GABA concentration increased significantly (16). It should be noted that there are no reports on the relationship between lactic fermentation and SPCs in vegetable juice.

In recent years, the relationship between gut microbiota and the gut–brain axis has attracted increasing attention (17, 18). The gut microbiota interferes with gastrointestinal physiology, metabolism, and immune function of hosts and affect the function and behavior of the central nervous system (CNS) (19–21). Bravo et al. found that anxiety and depressive behaviors were reduced in mice fed *Lacticaseibacillus rhamnosus*, but no changes in neurochemistry and behavior were found in mice with vagus nerve resection, suggesting the vagus nerve is the main communication route between gut microbiota and the brain (22). At present, a few diets have been shown to reduce anxiety-like behaviors and have physiological effects on the brain-gut axis (23). A recent study showed that schisandra chinensis improved anxiety- and depression-like behavior (24). Nevertheless, the effects of consuming vegetable-based fermentation juice rich in SPCs on sleep have not been studied.

This study aimed to investigate whether SPCs can be produced in FCJ by *Levilactobacillus brevis* (*L. brevis*) YSJ3. Furthermore, we investigated the effects of FCJ rich in SPCs on sleep. The results provide a theoretical basis for the application and development of SPCs-rich FCJ as a functional food.

## Materials and methods

### Preparation of fermented carrot juice by *Levilactobacillus brevis* YSJ3

Carrots were purchased from the local market (Provincial Academy of Agricultural Sciences vegetable farm, 200 Shiqiao Road, Hangzhou, Zhejiang, China). The carrots were cleaned, and roots were removed. They were cut into pieces and steamed in a steamer (Supor ZN28YK809-150, Zhejiang Supor Co., Ltd., Zhejiang, China). Then, using a 1:3 ratio of material to liquid (w/w), pure water was added, and the carrot was homogenized (high-speed blender; Ranbem-775, Guangdong, China). The carrot juice was filtered through a standard sample sieve with a diameter of 200 mm. Sugar (5% w/v; food grade) and sodium glutamate (0.5% w/v; food grade) were added. The mixture was stirred until dissolved, transferred to a 20-mL glass test tube, sealed, and sterilized in a vertical pressure steam sterilizer (YXQ-50SII, Shanghai Boxun Medical Biological Instrument Corp., China). The suitable condition for sterilization was 121°C high-pressure sterilization for 15 min (15, 25).

Current research suggested that *L. brevis* has good GABA synthesis ability (26). In our previous research, a high GABA-producing strain-*L. brevis* YSJ3 from Yeshanjun pickle (a traditional vegetable fermented food, Supplementary Figure 1) in Jingning She Autonomous County was screened. We found the GABA production from *L. brevis* YSJ3 reach about 760 μg/mL, which higher than the production of other *L. brevis* (Supplementary Table 1). Thus, *L. brevis* YSJ3 was selected as the fermentation stain. And *L. brevis* YSJ3 were deposited at the General Microbiology Center of China (CGMCC No.23307). The bacteria were identified by 16S rRNA gene sequence and subjected to whole genome sequencing using a combination of PacBio RS II Single Molecule Real Time (SMRT) and Illumina sequencing platforms. The genome of strain YSJ3 was deposited in NCBI under the accession numbers CP092264-CP092271. Chromosome genome maps of *L. brevis* YSJ3 chromosome are shown in Supplementary Figure 2. *L. brevis* YSJ3 were stored at −80°C in De Man, Rogosa and Sharpe (MRS, 20 g/L glucose, 4 g/L yeast extract, 10 g/L peptone, 8 g/L beef powder, 2 g/L dipotassium phosphate, 2 g/L ammonium citrate dibasic, 0.04 g/L manganese sulfate, 0.2 g/L magnesium sulfate, 1 mL/L tween-80, and 5 g/L sodium acetate) broth (CM1175, OXOID, Basingstoke, UK) containing 20% (v/v) glycerol (Sinopharm Chemical Reagent Co., Ltd., China) until use. *L. brevis* YSJ3 were propagated twice in MRS broth at 37°C for 30 h. Subsequently, the activated strain (8%, v/v) was inoculated in sterilized carrot juice and cultured at 37°C for 0, 12, 24, 36, and 48 h.

### Plate count, pH and lactic acid concentration

After 0, 12, 24, 36, and 48 h of culture, the cell viability of FCJ was determined by plate counting method (15). The sample was diluted with sterilized deionized water to obtain an appropriate cell density for measurement. Next, 100 μL of the sample was transferred to MRS agar (CM1163, OXOID, Basingstoke, UK) plates and incubated at 37°C for 48 h. The pH value of the sample was measured with a pH meter (METTLER TOLEDO, Zurich, Switzerland). Lactic acid concentration was tested by lactic acid content (LA) detection kit (Solatbio, Beijing, China).

### Analysis of short-chain fatty acids and neurotransmitters

#### Short-chain fatty acids

The SCFAs produced in FCJ were determined by gas chromatography-mass spectrometry (GC-MS; Agilent 5977B-7890B, Agilent Technologies, Palo Alto, CA, USA). The sample treatment method was modified according to a previously reported method (27). Carrot juice was fermented for 0, 12, 24, 36, and 48 h. The samples were centrifuged at 8,000 rpm for 20 min (Tabletop Centrifuge; Velocity 18R 5426026, Dynamica Scientific Ltd., Livingston, UK). Then, the supernatant was acidified by adding 50% concentrated sulfuric acid (Shanghai Lingfeng Chemical Reagent Co., Ltd., China) at a ratio of 24:1 (v/v). The mixture was kept at room temperature for 5 min and vortexed (Vortex finder; Scientific Industries, Inc., Bohemia, NY, USA) mixed once every 60 s. Then, the mixture was centrifuged at 5,000 × g for 10 min. The supernatant was combined with anhydrous ether (Shanghai Lingfeng Chemical Reagent Co., Ltd., China) at a ratio of 1:1 (v/v). The mixture was vortexed for 30 s and centrifuged at 5,000 × g for 10 min. Finally, the ether layer was collected and stored in a 2-mL injection bottle until further analysis. Acetic acid, propionic acid, isobutyric acid, butyric acid, isovaleric acid, and valeric acid were used as the standard solutions (Shanghai Macklin Biochemical Co., Ltd., China).

HP-FFAP column (30 m × 250 μm × 0.25 μm; Agilent Technologies, Palo Alto, CA, USA) was used for chromatographic analysis. The program to run was: 2 min at 90°C, increase to 150°C at 12°C/min, increase to 220°C at 20°C/min, and hold for 4.5 min. The run time was 15 min. Helium was the carrier gas, with a flow rate of 1 mL/min, and splitless mode was used for sample injection. The ion source, MS transfer line temperatures and mode selection were determined by reference to the reports (28, 29).

#### Neurotransmitters

High performance liquid chromatography-triple quadrupole mass spectrometry (LC-MS, SCIEX 5500 +, AB SCIEX, Massachusetts, USA) was used to determine the neurotransmitters produced in FCJ (30). First, the carrot juice samples fermented for 0, 12, 24, 36, and 48 h were centrifuged at 8,000 rpm for 20 min. The supernatant was combined with two times the volume of methanol (Merck KGaA, Darmstadt, Germany) and mixed via vortexing. The mixture was centrifuged at 12,000 rpm for 15 min at 4°C. The supernatant was collected and stored in a 2-mL injection bottle until further analysis. GABA (Shanghai Macklin Biochemical Co., Ltd., China) and 5-HT (Shanghai Aladdin Biochemical Technology Co., Ltd., China) were used as the standard solutions.

Chromatographic separation was performed using a ZORBAX Eclipse Plus C18 column (3.0 × 100 mm) with 1.8 μm particle size (Agilent Technologies Inc., Palo Alto, CA, USA). The injection volume was 1 μL. Eluent A consisted of ultra-pure water (Hangzhou Wahaha Group Co., Ltd., China) with 2 mM ammonium formate (Merck KGaA, Darmstadt, Germany) and 0.01% (v/v) formic acid (FA) (Merck KGaA, Darmstadt, Germany), and eluent B consisted of 100% methanol. The following gradient was applied: 0–0.5 min, 5% B; 0.5–5 min, 5–80% B; 5–6 min, 80% B; 6–6.1 min, 80–5% B; and 6.1–8 min, 5% B. The total runtime was 8 min, and the flow rate was 0.40 mL/min. The multiple reaction monitoring (MRM) transitions, declustering potential (DP), and collision energy (CE) are shown in Supplementary Table 2.

#### Animal studies and experimental design

**FIGURE 1:**
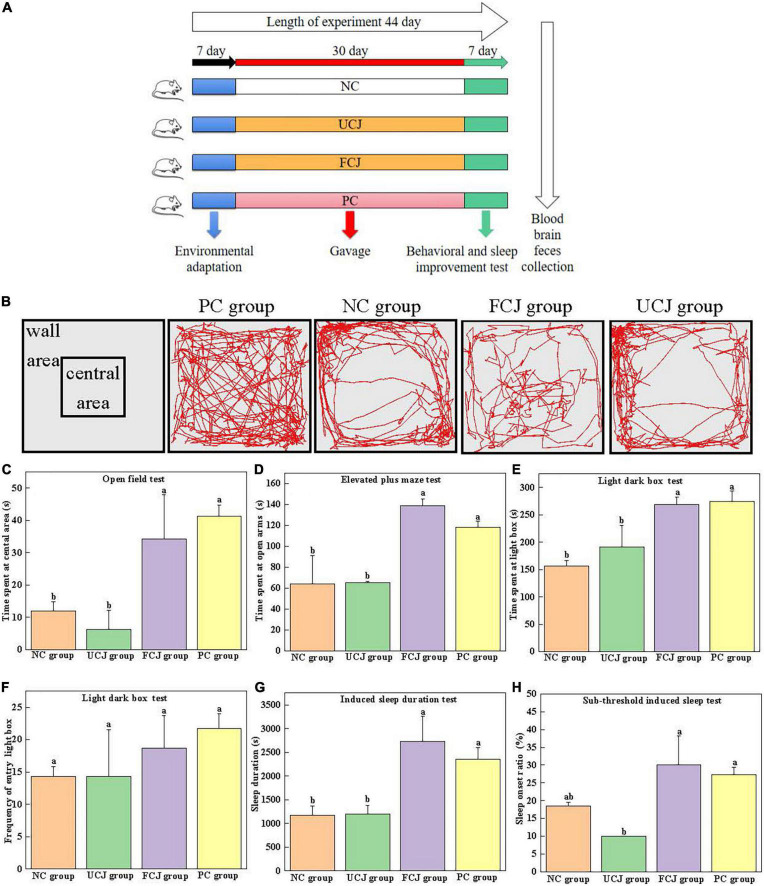
Effects of control (NC) group, unfermented carrot juice (UCJ) group, fermented carrot juice (FCJ) group, and positive control (PC) group on behavioral test and sleep improvement test in mice. **(A)** Schematic representation of the experimental procedure; **(B)** captured images for OFT; **(C)** the time spent at the central area for OFT; **(D)** the time spent at open arms for EPM; **(E)** the time spent at light box for LDB; **(F)** the frequency of entry into the light box for LDB; **(G)** sodium pentobarbital-induced sleep duration test; **(H)** sub-threshold sodium pentobarbital dose-induced sleep test. Data are mean ± SEM (*n* = 10). Different letters indicate significant difference (*p* < 0.05).

Specific pathogen free (SPF) ICR mice (male; 6 weeks of age) were purchased from Slack Laboratory Animal Co., Ltd. (Shanghai, China). Forty mice were randomly divided into four groups for treatment (*n* = 10): control (NC), unfermented carrot juice (UCJ), fermented carrot juice (FCJ), and positive control (PC) groups. Mice were placed in cages and subjected to a 12-h light/dark cycle in a room with temperature (23 ± 3°C) and humidity control (55 ± 15%). After acclimatization for 1 week, the mice in UCJ and FCJ group were orally given 10 mL/kg body weight (BW) of the UCJ and FCJ, respectively. The FCJ was fermented by *L. brevis* YSJ3 for 48 h. The number of viable bacteria was 10^8^ CFU/mL. The mice in NC group were received an equal volume of 0.85% aseptic normal salin by oral gavage. Meanwhile, the PC group was received daily doses (10 mg⋅kg^–1^⋅day^–1^) of GABA solution (1 mg/mL, Yuanye Biotechnology Co., LTD., Shanghai, China) by oral gavage. The dose of 10 mg⋅kg^–1^⋅day^–1^ GABA was equivalent to consumption of 48.65 mg GABA day^–1^ by a 60 kg human according to formula for dose translation (31). The process took place between 8:00 and 16:00 every day for 37 days. The animals underwent a series of behavioral tests and sleep improvement tests for 7 days before the end of the study. In each behavioral test, the experimenters treated each animal blindly. At the end of the experiment, the mice were fasted for 12 h, and anesthetized by ether and dissected. Blood was collected from the eyeballs and stored at 4°C for 4 h for coagulation. Then, the serum was obtained by centrifugation at 5,000 rpm at 4°C for 30 min. The mice were sacrificed by cervical dislocation, and the intestinal tract and intestinal contents were dissected. The mouse skull was cut open, and the brain was quickly separated, weighed, and immersed in liquid nitrogen. All serum, brain, and fecal samples were stored at -80°C until extracted for further testing. A schematic of the experimental process is shown in Figure 1A. The research was carried out in compliance with the International Guiding Principles for Biomedical Research Involving Animals. All experimental protocols were approved by the Animal Ethics Committee of Zhejiang Academy of Agricultural Sciences (Committee approval #2022ZAASLA47).

### Behavioral test

#### Open field test

The open field experimental box was a square box (50 × 50 × 40 cm). The color of the bottom was different from that of the mice to some extent. Open field tests (OFTs) were performed by following a modified protocol, as previously described (32–34). After 30 min of intragastric administration, each mouse was placed at the center of the device, and its activity within 5 min was recorded. After each mouse test, the instrument was washed with 75% ethanol to eliminate any possible odor cues. During the 5-min experiment, the movement routes and the time spent at the central area were recorded.

#### Light–dark box test

The light–dark box (LDB) consists of a light box and a dark box (100 × 50 × 50 cm), separated by a perforated partition (10 × 10 cm) in the middle. The light box provides a certain intensity of light. LDB tests were performed by following a modified protocol, as previously described (32). During the test, a mouse was gently placed in the central area of the light box in the LDB device, with its head facing the partition. The video recording system was used to track and record the behavioral performance of the mice in the LDB for 10 min. At the end of the test for each mouse, the instrument was cleaned with 75% alcohol before testing the next mouse. The time the mice spent in the light box and the frequency of entry into the light box were recorded. The mouse was considered to have completed the shuttle activity when its limbs completely entered the box from one side to the other.

#### Elevated plus maze test

The elevated plus maze (EPM) is composed of two open arms and two closed arms, each with a cross shape, suspended at a certain height. The length of the device was 70 cm, and the length of the arm was 30 cm. The width was 5 cm, and the height was 15 cm (32). The mouse was gently placed in the central platform region of the EPM device, with its head facing one of the open arms. The video recording system was used to track and record the behavioral performance of the mice in the EPM for 5 min. After each mouse was tested, the maze was cleaned with 75% alcohol before the next mouse was tested. The time the mice spent in the open arms was recorded. Mice were considered to be in the arm when their limbs were completely in one arm.

### Sleep improvement test

#### Sodium pentobarbital-induced sleep duration test

The dose of pentobarbital sodium intraperitoneal injection was preliminarily determined prior to the experiment. Thirty minutes after the last dose, each mouse was intraperitoneally injected 55 mg/kg.bw to induce sleep. The time from the disappearance of the righting reflex to the recurrence of the righting reflex was recorded as the sleep time of mice, and we recorded whether pentobarbital sodium prolonged the sleep duration in each group (34–37).

#### Sub-threshold sodium pentobarbital dose-induced sleep test

A preliminary experiment was conducted to determine the maximum subthreshold dose. Thirty minutes after the last dose, each mouse was intraperitoneally injected 40 mg/kg.bw to induce sleep. If the righting reflex of mice disappeared for more than 1 min, sleep was considered successful. The sleep onset ratio within 30 min was recorded (34–37).

#### Fecal deoxyribonucleic acid extraction and 16S rRNA gene sequencing

Fecal genomic deoxyribonucleic acid (DNA) was extracted using DNA Kit (Omega Bio-tek, Norcross, GA, USA) according to the manufacturer’s instructions. 16S rRNA gene sequencing by the hypervariable regions V3–V4 (primer pairs 338F 5-ACTCCTACGGGAGGCAGCAG-3, and 806R 5-GGACTACH-VGGGTWTCTAAT-3) were performed by Shanghai Majorbio Bio-pharm Technology Co., Ltd. (Shanghai, China) using Illumina MiSeq platform (Illumina, San Diego, USA). The data processing and analysis is described in detail in Supplementary Methods.

#### Determination of short-chain fatty acids in mice feces

Colon content samples were collected and determined by GC-MS (27). Briefly, colon content particles (0.1 g) were mixed with 1,200 μL ultrapure water, mashed, and swirled for 1 min. To the suspension, 50 μL of 50% sulfuric acid was added, and the mixture was kept at room temperature for 5 min and mixed every 60 s. After the sample was centrifuged at 5,000 × g for 10 min, the supernatant (500 μL) was transferred to a 1.5-mL centrifuge tube, and 500 μL anhydrous ether was added for 30 s. The sample was centrifuged at 5,000 × g for 10 min, and the upper ether layer was collected for GC-MS analysis. The instrumental analysis method was the same as described above.

#### Determination of neurotransmitters in mice feces, brain, and serum

The mice were killed by removing cervical vertebra, and the entire brain was removed by ice bath craniotomy. After the brain was weighed, ultra-pure water was added to the homogenate at the ratio of 1:1 (m/v), and it underwent ultrasonication for 10 s. Then, trifluoroacetic acid (TFA) (Macklin, China) was added in a volume ratio 5:1, and the protein was precipitated by whirlpool mixing. Next, the supernatant was centrifuged at 12,000 rpm for 20 min at 4°C and analyzed by LC-MS. The preparation method for serum and fecal samples was the same as that of the brain homogenate. GABA, 5-HT, 5-hydroxyindole-3-acetic acid (5-HIAA, Shanghai Aladdin Biochemical Technology Co., Ltd., China), dopamine (DA, Shanghai Macklin Biochemical Co., Ltd., China), and norepinephrine (NE, Shanghai Macklin Biochemical Co., Ltd., China) were used as the standard solutions. The instrumental analysis method was the same as described above.

### Statistical analysis

Statistical analysis was performed using GraphPad Prism 9.0 software and SPSS 19.0 software. All data are expressed as mean ± standard deviation. All data in Table 1 were carried out with at least three replicates, and repeated measures ANOVA was used to analyze significance.

**TABLE 1:** *L. brevis* YSJ3 growth, pH, lactic acid, short chain fatty acids, and neurotransmitters in fermented carrot juice at different fermented time.

Fermented carrot juice		Fermentation time (h)				
		0	12	24	36	48
*L. brevis* YSJ3 growth (×10^8^ CFU/mL)		—	2.42 ± 0.22^a^	2.67 ± 0.14^a^	2.23 ± 0.19^a^	2.20 ± 0.03^a^
Fermentation Ph		4.36 ± 0.04^d^	4.16 ± 0.04^c^	4.07 ± 0.03^b^	3.87 ± 0.05^ab^	3.78 ± 0.03^a^
Lactic acid (mg/L)		222.42 ± 1.20^c^	1990.05 ± 55.38^b^	2884.90 ± 442.93^ab^	3543.46 ± 209.47^a^	4322.44 ± 173.13^a^
Short chain fatty acids (mg/L)	Acetic acid	—	3865.66 ± 132.31^b^	4182.91 ± 449.56^ab^	4835.45 ± 12.44^a^	4912.14 ± 15.14^a^
	Propionic acid	—	—	—	—	—
	Isobutyric acid	—	—	—	—	—
	Butyric acid	39.30 ± 0.54^c^	43.41 ± 1.12^b^	41.83 ± 0.45^b^	46.29 ± 0.67^a^	42.50 ± 1.39^abc^
	Isovaleric acid	—	17.15 ± 0.47^c^	17.19 ± 0.46^abc^	17.73 ± 0.34^b^	18.68 ± 0.44^a^
	Valeric acid	—	—	—	—	—
Sleep-promoting neurotransmitters (mg/L)	GABA	0.52 ± 0.01^d^	27.58 ± 0.05^c^	60.89 ± 0.10^b^	71.09 ± 0.32^a^	74.08 ± 0.28^a^
	5-HT	—	—	—	—	—

“—” means not detected. Different lowercases indicate significant differences (*p* < 0.05). All data in this table were carried out with three replicates, and expressed as mean ± standard deviation.

In mice experiments, *a priori* sample size calculations were not conducted. Behavioral test and sleep improvement test were carried out in 10 mice (*n* = 10). In addition, for the detection and analysis of gut microbes, four samples were used (*n* = 4). And each sample were collected from 2 to 3 mice (e.g., the fecal from the first and second mice was gathered together as the first sample). For the concentration of SCFAs in mice feces and the concentration of neurotransmitters in mice feces, brain, and serum, six mice were randomly selected from each experimental group (*n* = 6). All data were performed with a normal distribution (Shapiro-Wilk test). If assumptions were met normal distribution, one-way ANOVA were performed with Tukey’ multiple comparisons test. If assumptions were not met normal distribution, non-parametric test (Kruskal-Wallis) were performed with Dunn’s multiple comparisons test. The *p*-value of multiple comparisons (*p*_*adj*_) was adjusted by family-wise significance and confidence levels of 0.05 (95% confidence interval). Statistical significance was acknowledged when *p*-values were below 0.05 (*p* < 0.05).

## Results

### Growth of *Levilactobacillus brevis* YSJ3 in fermented carrot juice

The cell viability of *L. brevis* YSJ3 in carrot juice after 12, 24, 36, and 48 h of fermentation was measured (Table 1). The bacteria were not detected in UCJ, which was used as the control group to evaluate the growth of *L. brevis* YSJ3. As the fermentation time increased, the colony numbers of *L. brevis* YSJ3 also increased. The fastest growth rate was observed from 0 to 12 h. When the fermentation time was 12 h, *L. brevis* YSJ3 entered a stable growth stage. The viable count of *L. brevis* YSJ3 remained around 2 × 10^8^ CFU/mL. This indicates that *L. brevis* YSJ3 can grow in carrot juice due to its sugar content, which provides adequate energy and a carbon source for the bacteria. Fuente et al. and Multari et al. found that lactic acid bacteria could grow and live in plant-based fermented juices (15, 38). In addition, Table 1 shows that the pH of the FCJ decreased with fermentation time, and the lowest pH (3.78) was reached after 48 h of fermentation. On the contrary, the level of lactic acid increased with fermentation time. Thus, the higher bacterial growth decreases the pH and increases LA *via* organic acid metabolism, such as lactic acid and acetic acid. This result is in agreement with previously reported results by Hashemi et al. (39). Therefore, carrot juice rich in sugar is a suitable medium for the growth of lactic acid bacteria.

### Sleep-promoting compounds in fermented carrot juice by *Levilactobacillus brevis* YSJ3

Certain probiotic strains (mainly lactic acid bacteria) are known to produce SCFAs and sleep-promoting neurotransmitters, GABA and 5-HT, by fermentation (14, 26). For this reason, these compounds were identified in *L. brevis* YSJ3 FCJ at different fermentation stages. The SCFAs concentration in FCJ at 12, 24, 36, and 48 h was determined by GC-MS (Table 1). Only butyric acid was detected in the UCJ, at a concentration of 39.30 ± 0.54 mg/L. As fermentation progressed, the content of acetic acid, isovaleric acid, and butyric acid significantly increased. The maximum concentration of acetic acid, isovaleric acid, and butyric acid reached 4912.14 ± 15.14, 18.68 ± 0.44, and 46.29 ± 0.67 mg/L, respectively. In contrast, propionic acid, isobutyric acid, and valeric acid were not detected in the FCJ. These results indicate that *L. brevis* YSJ3 in carrot juice produced acetic acid, isovaleric acid, and butyric acid, and these three fatty acids are typical metabolic products of organic acid metabolism by *L. brevis*. GABA and 5-HT concentrations in FCJ at 0, 12, 24, 36, and 48 h fermentation were determined by LC-MS (Table 1). No 5-HT was detected in unfermented and FCJ. However, GABA content significantly increased with increasing fermentation time from 0.52 ± 0.01 to 74.08 ± 0.28 mg/L. Therefore, *L. brevis* YSJ3 FCJ was rich in SCFAs and GABA, which would effectively improve sleep.

### Behavioral effect of sleep-promoting components-enriched fermented carrot juice on mice

BW of mice showed no statistically significant differences among all the groups (Supplementary Table 3). OFT, LDB, and EPM tests are widely used for detecting anxiety-like behavior in animals (40). The captured images for OFT show the FCJ and PC groups were more frequently entered the central area then the UCJ and NC groups (Figure 1B). And the images of FCJ group and PC group were not different. It demonstrated that mice in FCJ group had a similar effect to mice in PC group. In addition, the FCJ and PC groups spent significantly more time in the central area (34.25 ± 13.67 and 41.25 ± 3.5 s, respectively), compared with the UCJ group (6.25 ± 5.85 s) and NC group (12.00 ± 2.94 s) (Figure 1C). Similarly, EPM and LDB tests showed that the FCJ and PC groups spent significantly more time in the open arms and light box (Figures 1D–F). Furthermore, the effects of FCJ, UCJ, PC, and NC treatments on sodium pentobarbital-induced sleeping behaviors are shown in Figure 1G. Sleep duration more than doubled after oral administration of FCJ and GABA compared to UCJ and saline. The sleep onset ratio of the FCJ and PC groups was 30 and 27%, respectively (Figure 1H). This represents a significant increase, compared with the UCJ groups (10%). These results demonstrate that SPCs-enriched FCJ could effectively relieve anxiety and improve sleep.

### The analysis of mice gut microbiota in four groups

**FIGURE 2:**
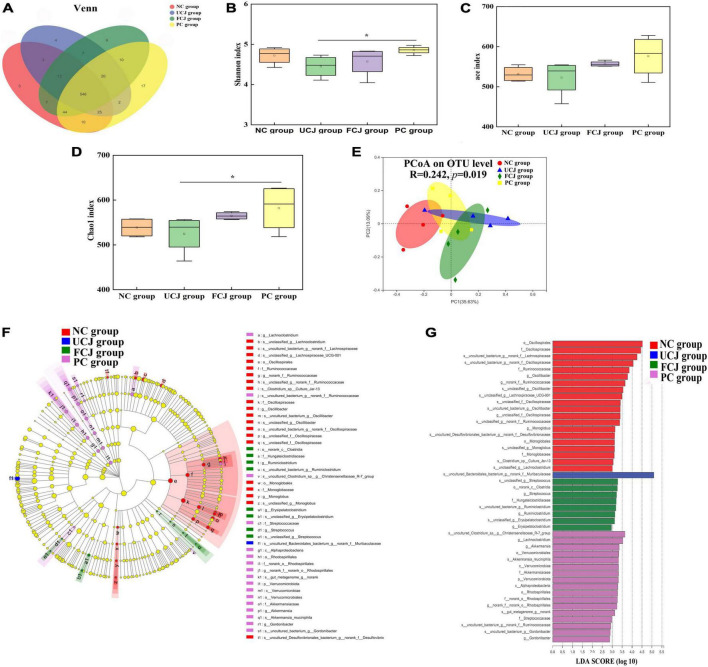
Control (NC), unfermented carrot juice (UCJ), fermented carrot juice (FCJ), and positive control (PC) group gut microbiota composition (*n* = 4). **(A)** Venn diagram of OTU in the feces; **(B)** Shannon diversity indexes; **(C)** Ace diversity indexes; **(D)** Chao 1 diversity indexes; **(E)** PCoA analysis; **(F)** LEfSe multi-level species difference discriminant analysis; **(G)** linear discriminant analysis. *p < 0.05 was considered to indicate significant difference.

To assess the effect of FCJ on gut microbiota composition, the mice feces was detected by bacterial 16S rRNA sequencing. In the Venn diagram, the number of OTUs was not significantly different in all groups, and most OTUs were shared (Figure 2A). In addition, the α diversity indices for the UCJ and FCJ groups, including Shannon, Ace, and Chao1, were not significantly different, compared to the NC group (Figures 2B–D). This suggests that the microbial richness and diversity did not change after oral administration of normal saline and fermented and UCJ. In addition, PCoA shows that the beta diversity of the FCJ group was separated from the NC group (Figure 2E). But, the FCJ group and UCJ group did not differ. This indicates that some ingredients in carrot juice may change the composition and structure of mice gut microbiota. To identify specific taxa, relative abundances were assessed at the phylum and genus levels (Supplementary Tables 4, 5). Interestingly, *Ruminiclostridium* and *Akkermansia* found in the FCJ group and PC group, respectively, which was significantly higher than that in the NC group and UCJ group, as indicated by LEfSe (LDA Effect Size) multi-level species difference discriminant analysis and linear discriminant analysis (LDA) (Figures 2F,G).

### Sleep-promoting components-rich fermented carrot juice improved the content of short-chain fatty acids in mice gut

**FIGURE 3:**
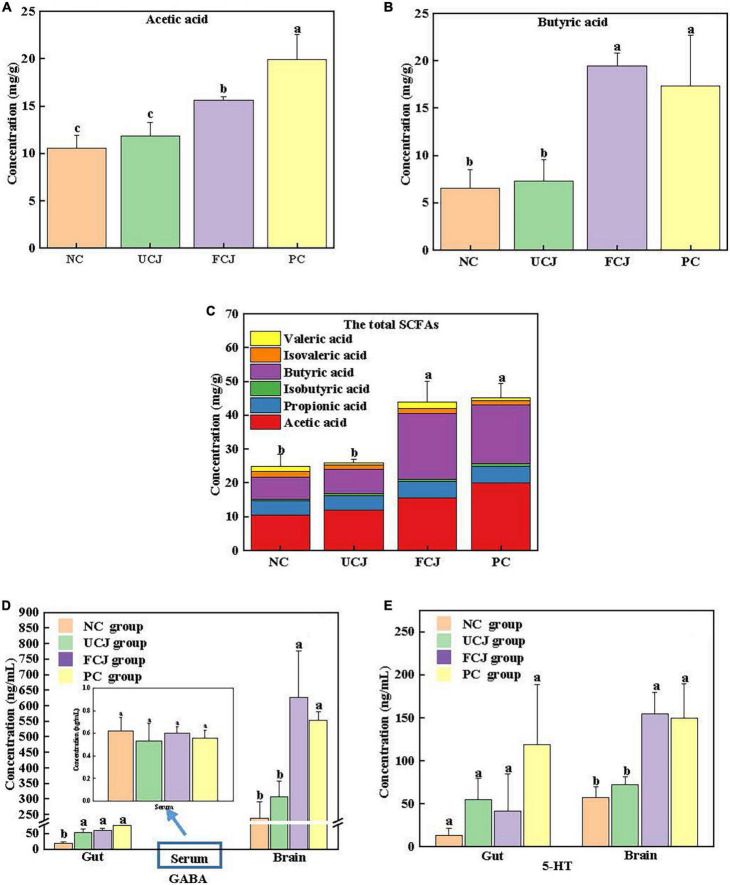
The content of SCFAs and neurotransmitters in mice gut, serum, and brain. **(A)** The content of acetic acid in gut; **(B)** the content of butyric acid in gut; **(C)** the content of total SCFAs in gut; **(D)** the content of GABA in mice gut, serum, and brain; **(E)** the content of 5-HT in mice gut and brain. Different letters indicate significant difference (*p* < 0.05). Six mice were randomly selected from each experimental group (*n* = 6).

Currently, there is growing evidence linking changes in gut microbiota to gut metabolic changes (9). In particular, SCFAs can be produced by gut microbiota. Thus, acetic acid, propionic acid, isobutyric acid, butyric acid, isovaleric acid, and valeric acid were detected in mice colon content by GC-MS. The concentrations of acetic acid and butyric acid were higher than that of other SCFAs, and they observably improved in the FCJ and PC groups, compared to the NC and UCJ groups (Figures 3A,B). The concentration of propanoic acid, isobutyric acid, valeric acid, and isovaleric acid was not significantly different among the four groups. In addition, the total concentration of SCFAs in the FCJ and PC groups reached 43.83 and 45.06 mg/g, respectively, which were significantly higher than 24.85 and 25.93 mg/g in the NC and UCJ groups, respectively (Figure 3C).

### Neurotransmitter levels in mice gut, serum, and brain

The content of neurotransmitters, such as GABA, 5-HT, 5-HIAA, DA, and NE, affects sleep quality and quantity (41). These five neurotransmitters were measured in mice gut and brain by LC-MS. Figures 3D,E show that the levels of GABA and 5-HT significantly increased in the brain after oral administration of GABA and FCJ. It is worth noting that the GABA content was not significantly different in the serum of all groups. The content was only about 0.6 ng/mL. Thus, GABA in the gut was possible not absorbed by the intestine into the bloodstream. But there was another possibility that the time of measurement may be missed in circulating GABA concentrations. In addition, the level of 5-HIAA, DA, and NE was not significantly different in mice gut and brain between the four groups (Supplementary Table 6).

## Discussion

Increasing evidence suggests that the incorporation of probiotics, SCFAs, and neurotransmitters as dietary supplements can mitigate insomnia and promote sleep quality or quantity (8, 22, 42). In our research, an SPCs-rich *L. brevis* YSJ3 FCJ by was developed to improve sleep function. *L. brevis* YSJ3 could increase the content of SCFAs and sleep-promoting neurotransmitters, such as acetic acid, isovaleric acid, butyric acid, and GABA. *L. brevis* are considered to be an SCFAs-producing strain via organic acid metabolism (26). In addition, *L. brevis* exhibited a strong ability to produce GABA, compared to other lactic acid bacteria (34). These GABA-producing bacteria synthesize GABA mainly via the glutamate decarboxylase (GAD) system (43, 44). The entire genome of *L. brevis* YSJ3 had been sequenced in our previous research (Supplementary Figure 2). The results of gene prediction show that *L. brevis* YSJ3 had a complete set of gad operons, including GadA/B, GadC, and GadR (Supplementary Figure 3). Furthermore, we proved that *L. brevis* YSJ3 can produce GABA *in vitro* from MRS broth (Supplementary Figure 4). Therefore, GABA can be produced and accumulated in FCJ *via L. brevis* YSJ3 fermentation. More importantly, mice treated with daily supplementation of SPCs-rich FCJ increased their entry frequency and time spent at the central area, open arms, and light box. Additionally, sleep improvement tests show that sleep duration of mice improved significantly after supplementation with FCJ.

The latest studies have suggested that certain dietary foods and supplements containing prebiotics, probiotics, and SPCs can improve sleep by affecting microbiota composition (24, 27). We compared the composition and diversity of bacterial communities from mice fecal samples from the FCJ, UCJ, PC, and NC groups. Alpha diversity analysis revealed that microbial richness and diversity did not change upon oral administration of GABA, FCJ, and UCJ. Similar results were reported by Song et al. and Tang et al. (41, 45). However, the beta diversity of the FCJ group was distinguished from that of the NC group and overlapped with that of the UCJ group, suggesting that some ingredients in carrot juice may change the composition and structure of mice gut microbiota. At the genus level, the relative abundances of *Ruminiclostridium* and *Akkermansia* significantly increased in the FCJ group and PC group, respectively, compared with those in the NC group and UCJ group. It is worth noting that these genera are all SCFAs-producing bacteria. Changes in probiotic levels could alter the ability to produce SCFAs. In this study, the fecal SCFAs level of the FCJ group was markedly higher than that of the NC and UCJ groups, especially acetic acid and butyric acid. Interestingly, butyrate has a positive effect on sleep via a sensory mechanism (42). Moreover, acetate has been shown to modulate the production of gut neurotransmitters (46). The high level of gut acetic acid and butyric acid was partly food-derived and partly from SCFAs-producing gut bacteria. Thus, consumption of foods rich in acetic acid and butyric acid can effectively improve the level of SCFAs in the gut.

GABA, a SPCs, can inhibit arousal systems to promote sleep. In general, GABA interacts through the vagus nerve and the production of other neurotransmitters to affect sleep (34, 47). In addition, some reports have proven that GABA can cross the blood brain barrier (48). Thus, we determined the content of GABA in the gut, serum, and brain of mice in all groups. The content of GABA in the gut and brain of the FCJ and PC groups was significantly increased compared with the NC group. However, a low level of GABA was found in mice serum, and no significant difference was determined for all groups. We speculated that gut-GABA may not interfere with sleep by crossing the blood-brain barrier. But the reasons need to be further verified. Indeed, intestinal tissues and vagal afferent neurons have a variety of GABA receptors, such as ionotropics GABA_*A*_ receptor and metabotropic GABA_*B*_ receptors (49). GABA can bind to these receptors to modulate the production and secretion of other neurotransmitters in the gut and brain. Our results revealed that only 5-HT in the brain of mice substantially increased after oral administration of FCJ and GABA. However, the level of 5-HIAA, DA, and NE was not significantly different in mice gut and brain. Many reports have suggested that probiotic administration, especially *Lactobacillus* and *Bifidobacterium*, can also improve sleep (31, 50–53). These strains perform functional communication by changing gut microbiota composition to increase the levels of several sleep-promoting metabolites (17, 22). Furthermore, these gut metabolites exert their effects through the vagus nerve (17). Therefore, oral administration of GABA and the consumption of *L. brevis* YSJ3-rich foods contribute to the improvement of sleep. But, the detailed mechanism is not clear, which needs further validation. In this study, we only have focused on the effects of SCFAs, GABA, and probiotics on sleep. However, FCJ also contain other substances, which may be related to sleep. In addition, this is a limitation of this paper in sample size and mixed fecal samples for gut microbiota analysis. In the further, a large sample size of gut microbiota analysis is required. Furthermore, detecting neurotransmitter levels at single time points may affect the accuracy of the results and conclusions. Therefore, the promoting sleep mechanisms of carrot-based fermentation juice need to be more detailed and in-depth studied.

## Conclusion

In this work, the contents of SPCs (acetic acid, isovaleric acid, butyric acid, and GABA) increased significantly in carrot juice fermented by *L. brevis* YSJ3. The results of OFT, LDB, EPM, and sleep improvement tests show that oral administration of FCJ improved anxiety and sleep of mice. Moreover, the levels of SCFAs and GABA in the gut, as well as GABA and 5-HT in the brain, were significantly increased in the FCJ group, compared with NC group. The results support that SPCs-enriched FCJ has potential functionality to improve sleep. This probably linked to the fermentation of carrot juice and the compounds produced during the fermentation.

## Data availability statement

The original contributions presented in the study are included in the article/Supplementary material, further inquiries can be directed to the corresponding author/s. The data presented in the study are deposited in the NCBI repository, accession number PRJNA903512.

## Ethics statement

This animal study was reviewed and approved by the Animal Ethics Committee of Zhejiang Academy of Agricultural Sciences (Committee approval #2022ZAASLA47).

## Author contributions

DYL and JZ prepared the fermented carrot juice, sleep-promoting components test, the animal experiments, and significantly contributed to writing the manuscript. JC performed all animal experiment. CZ was responsible for data processing and article revision. HY and DQL were responsible for designing and managing the entire study. All authors read and approved the final manuscript.
