# Supplementary material for: Carrot-based fermentation juice rich in sleep-promoting components improved sleep in mice
Source: Front Nutr. 2022 Nov 29;9:1043055. doi: 10.3389/fnut.2022.1043055 (PMC9745110; doi:10.3389/fnut.2022.1043055)
Supplement: Supplementary file 1 [file Data_Sheet_1.docx]

**Supplemental methods**

**Data processing**

Raw FASTQ files were de-multiplexed using an in-house perl script, and then quality-filtered by fastp version 0.19.6 and merged by FLASH version 1.2.7 with the following criteria: (i) the 300 bp reads were truncated at any site receiving an average quality score of <20 over a 50 bp sliding window, and the truncated reads shorter than 50 bp were discarded, reads containing ambiguous characters were also discarded; (ii) only overlapping sequences longer than 10 bp were assembled according to their overlapped sequence. The maximum mismatch ratio of overlap region is 0.2. Reads that could not be assembled were discarded; (iii) Samples were distinguished according to the barcode and primers, and the sequence direction was adjusted, exact barcode matching, 2 nucleotide mismatch in primer matching. Then the optimized sequences were clustered into operational taxonomic units (OTUs) using UPARSE 7.1 with 97% sequence similarity level. The most abundant sequence for each OTU was selected as a representative sequence. The OTU table was manually filtered, i.e., chloroplast sequences in all samples were removed. To minimize the effects of sequencing depth on alpha and beta diversity measure, the number of 16S rRNA gene sequences from each sample were rarefied to 20,000, which still yielded an average Good’s coverage of 99.09% , respectively.

**Statistical Analysis**

Bioinformatic analysis of the gut microbiota was carried out using the Majorbio Cloud platform (<https://cloud.majorbio.com>). Based on the OTUs information, Venn diagram were performed on R software (version 3.3.1). Alpha diversity indices including observed OTUs, Shannon, Ace, and Chao1 were calculated with Mothur v1.30.2. Principal co-ordinates analysis (PCoA) were performed by Bray-curtis dissimilarity using R software (version 3.3.1). Community composition analysis (Bar picture) were performed by R software (version 3.3.1). The linear discriminant analysis (LDA) effect size (LEfSe) (http://huttenhower.sph.harvard.edu/LEfSe) was performed to identify the significantly abundant taxa (phylum to genera) of bacteria among the different groups (LDA score > 3, *p* < 0.05).

**Supplementary Table**

**Table S1 Production of GABA by *Levilactobacillus brevis* isolated from different fermented foods**

| Number | Strain number | GABA content (mg/mL) | Foodstuff |
| --- | --- | --- | --- |
| 1 | YSJ-1 | 0.3575 | Yeshanjun pickle |
| 2 | YSJ-2 | 0.3511 | Yeshanjun pickle |
| 3 | YSJ-3 | 0.7677 | Yeshanjun pickle |
| 4 | YSJ-4 | 0.3365 | Yeshanjun pickle |
| 5 | YSJ-5 | 0.0905 | Yeshanjun pickle |
| 6 | YSJ-6 | 0.2853 | Yeshanjun pickle |
| 7 | HZPS-4 | 0.2056 | Paosun pickle |
| 8 | HZPC2-1 | 0.1994 | Paocai pickle |
| 9 | LB-1 | 0.0220 | Fermented milk |
| 10 | LB-2 | 0.0655 | Fermented milk |
| 11 | LB-3 | 0.0250 | Fermented milk |
| 12 | LB-4 | 0.0292 | Fermented milk |
| 13 | LB-5 | 0.1411 | Fermented milk |
| 14 | HZXC-3 | 0.0032 | Xuecai |
| 15 | HZXC-2 | 0.0031 | Xuecai |
| 16 | HZZC-3 | 0.0030 | Zhacai |

| **Table S2 Multiple reaction monitoring conditions of each analyte in positive ionization mode** | | | | |
| --- | --- | --- | --- | --- |
| Analyte | Precursor ion (m/z) | Product ion (m/z) | Declustering potential (volts) | Collision energy (volts) |
| γ-Aminobutyic acid | 104.0 | 69.0 | 50 | 23 |
| 5-Hydroxytryptamine | 177.0 | 160.0 | 48 | 18 |
| 5-Hydroxyindole-3-acetic Acid | 192.0 | 145.9 | 90 | 23 |
| Dopamine | 154.0 | 91.0 | 40 | 31 |
| Norepinephrine | 170.1 | 107.2 | 10 | 28 |

**Table S3 The weight of mouse (g)**

| group | 0d | 1d | 2d | 3d | 6d | 8d | 11d | 13d | 15d | 18d | 21d | 24d | 27d | 30d | 33d | 36d | 39d | 40d | 43d |
| --- | --- | --- | --- | --- | --- | --- | --- | --- | --- | --- | --- | --- | --- | --- | --- | --- | --- | --- | --- |
| NC group | 23.88±0.87 | 26.71±1.23 | 27.59±1.28 | 28.31±1.07 | 29.60±1.59 | 31.52±1.24 | 32.37±1.29 | 32.68±1.57 | 33.07±1.68 | 33.61±1.64 | 33.98±1.60 | 34.05±1.85 | 35.06±1.53 | 34.95±1.77 | 35.25±1.66 | 35.19±1.90 | 36.55±1.80 | 36.45±1.65 | 36.89±1.72 |
| UCJ group | 23.65±1.24 | 26.83±1.24 | 27.42±1.46 | 28.1±1.58 | 29.45±1.96 | 31.65±2.10 | 32.22±1.96 | 32.92±2.20 | 33.46±2.24 | 34.04±2.26 | 34.13±2.37 | 34.58±2.20 | 35.57±2.33 | 35.54±2.39 | 35.82±2.38 | 35.5±2.39 | 36.73±2.30 | 36.52±2.49 | 37.13±2.68 |
| FCJ group | 24.14±1.63 | 27.22±2.20 | 27.52±1.82 | 28.41±2.02 | 29.81±1.75 | 31.77±1.72 | 32.74±1.73 | 33.03±1.87 | 33.57±1.84 | 33.98±1.94 | 34.09±1.91 | 34.57±2.13 | 35.3±2.17 | 35.33±2.00 | 35.47±2.08 | 35.95±1.89 | 36.35±2.19 | 36.52±2.10 | 36.81±2.31 |
| PC group | 23.27±0.94 | 26.84±1.69 | 26.82±1.68 | 27.61±1.73 | 29.69±2.23 | 31.53±2.32 | 32.48±1.99 | 32.91±1.98 | 33.59±1.89 | 34.15±1.94 | 34.43±2.00 | 34.47±2.29 | 35.42±2.21 | 35.41±1.96 | 35.63±1.86 | 36.36±2.11 | 36.90±1.98 | 36.76±2.08 | 37.28±2.35 |

| **Table S4 The relative abundance (%) of bacterial community in NC, UCJ, FCJ and PC groups at phylum level** | | | | |
| --- | --- | --- | --- | --- |
| samples | NC | UCJ | FCJ | PC |
| Bacteroidota | 34.26±17.37% | 63.38±22.34% | 55.53±16.56% | 51.73±13.53% |
| Firmicutes | 61.05±16.19% | 31.51±20.27% | 38.06±13.41% | 42.96±14.32% |
| Campilobacterota | 1.70±1.21% | 1.72±1.45% | 1.79±1.56% | 1.40±0.37% |
| Deferribacterota | 1.15±1.28% | 1.39±1.70% | 1.82±1.96% | 1.05±1.30% |
| Patescibacteria | 0.61±0.52% | 0.23±0.35% | 1.10±0.66% | 0.44±0.57% |
| Cyanobacteria | 0.18±0.07% | 0.35±0.45% | 0.58±0.42% | 0.87±0.50% |
| Proteobacteria | 0.20±0.37% | 0.62±0.49% | 0.25±0.17% | 0.46±0.27% |
| others | 0.84±0.39% | 0.80±0.30% | 0.88±0.24% | 1.09±0.52% |

| **Table S5 The relative abundance (%) of bacterial community in NC, UCJ, FCJ and PC groups at genus level** | | | | |
| --- | --- | --- | --- | --- |
| samples | NC | UCJ | FCJ | PC |
| Muribaculaceae | 15.88±10.81% | 52.97±26.58% | 40.10±17.15% | 34.90±11.37% |
| Lachnospiraceae | 39.06±13.71% | 16.85±14.57% | 14.17±7.98% | 25.52±10.70% |
| Lactobacillus | 3.46±2.25% | 5.40±2.29% | 14.69±15.32% | 4.74±2.76% |
| Alistipes | 9.96±8.41% | 3.54±1.71% | 6.56±3.23% | 6.80±2.26% |
| Bacteroides | 2.87±4.01% | 1.69±1.76% | 2.25±0.61% | 2.66±0.49% |
| Helicobacter | 1.70±1.21% | 1.72±1.45% | 1.79±1.56% | 1.40±0.37% |
| Oscillospiraceae | 3.23±1.35% | 1.15±1.17% | 0.86±0.29% | 1.47±0.65% |
| Mucispirillum | 1.15±1.28% | 1.39±1.70% | 1.82±1.96% | 1.05±1.30% |
| Rikenella | 1.94±1.18% | 0.71±0.41% | 1.27±0.83% | 1.42±0.33% |
| Prevotellaceae | 0.47±0.38% | 0.99±1.16% | 1.89±2.00% | 1.48±0.78% |
| Odoribacter | 1.58±0.98% | 0.69±0.23% | 1.09±0.41% | 0.96±0.54% |
| Clostridia | 1.06±0.79% | 0.70±1.10% | 0.77±0.45% | 1.49±2.01% |
| Roseburia | 1.49±0.39% | 0.99±0.67% | 0.87±0.67% | 0.81±0.07% |
| Colidextribacter | 1.64±1.12% | 0.71±0.56% | 0.49±0.16% | 0.82±0.36% |
| Clostridia | 1.06±0.96% | 0.76±0.20% | 0.52±0.40% | 1.08±0.57% |
| Parabacteroides | 0.46±0.73% | 0.54±0.15% | 0.70±0.28% | 1.50±1.03% |
| Ruminococcaceae | 1.32±0.32% | 0.47±0.35% | 0.55±0.34% | 0.76±0.34% |
| Lachnoclostridium | 0.83±0.22% | 0.38±0.27% | 0.56±0.17% | 0.97±0.35% |
| Oscillibacter | 1.42±0.44% | 0.38±0.37% | 0.29±0.09% | 0.54±0.26% |
| Candidatus | 0.61±0.52% | 0.23±0.35% | 1.10±0.66% | 0.44±0.57% |
| Muribaculum | 0.25±0.20% | 1.12±0.69% | 0.49±0.36% | 0.50±0.25% |
| Gastranaerophilales | 0.18±0.07% | 0.35±0.45% | 0.58±0.42% | 0.87±0.50% |
| Rikenellaceae | 0.30±0.40% | 0.24±0.04% | 0.35±0.29% | 0.61±0.44% |
| Eubacterium | 0.73±0.79% | 0.21±0.22% | 0.38±0.18% | 0.21±0.11% |
| Parasutterella | 0.15±0.28% | 0.53±0.47% | 0.20±0.17% | 0.07±0.09% |
| Faecalibaculum | 0.00±0.01% | 0.68±1.20% | 0.04±0.04% | 0.23±0.27% |
| others | 7.20±1.21% | 4.63±2.62% | 5.64±0.62% | 6.70±1.28% |

| **Table S6 The content of GABA, 5-HT, 5-HIAA, DA and NE in mice gut and brain (ng/mL)** | | | | | | | | | | |
| --- | --- | --- | --- | --- | --- | --- | --- | --- | --- | --- |
|  | GABA | | 5-HT | | 5-HIAA | | DA | | NE | |
|  | gut | brain | gut | brain | gut | brain | gut | brain | gut | brain |
| NC | 18.96±4.41 | 237.03±53.99 | 13.30±8.39 | 57.02±33.46 | 193.24±75.32 | 328.87±32.59 | 73.40±33.60 | 155.07±86.27 | 66.32±30.52 | 12.44±1.53 |
| UCJ | 53.06±11.85 | 307.10±50.53 | 54.73±24.98 | 72.51±32.87 | 166.79±33.41 | 325.23±10.69 | 77.13±12.15 | 178.71±71.10 | 51.67±13.51 | 12.57±1.25 |
| FCJ | 60.18±5.50 | 626.97±149.16 | 41.69±43.29 | 153.67±97.79 | 169.35±174.05 | 310.85±32.48 | 65.61±39.71 | 376.15±247.19 | 49.12±37.64 | 12.58±0.75 |
| PC | 76.11±16.97 | 552.87±26.74 | 118.92±70.01 | 148.04±45.58 | 151.17±66.66 | 301.03±42.67 | 64.66±5.67 | 268.91±110.34 | 52.95±12.24 | 12.52±1.21 |


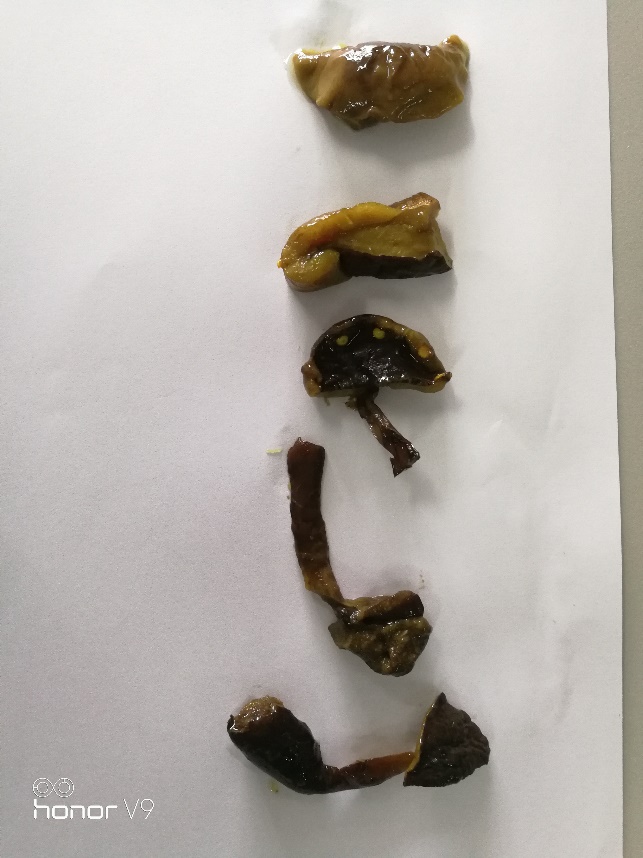
**Supplementary Figure**

**Supplementary Fig.S1.** Yeshanjun pickle, a traditional vegetable fermented food from Jingning She Autonomous County, Zhejiang Province, China.


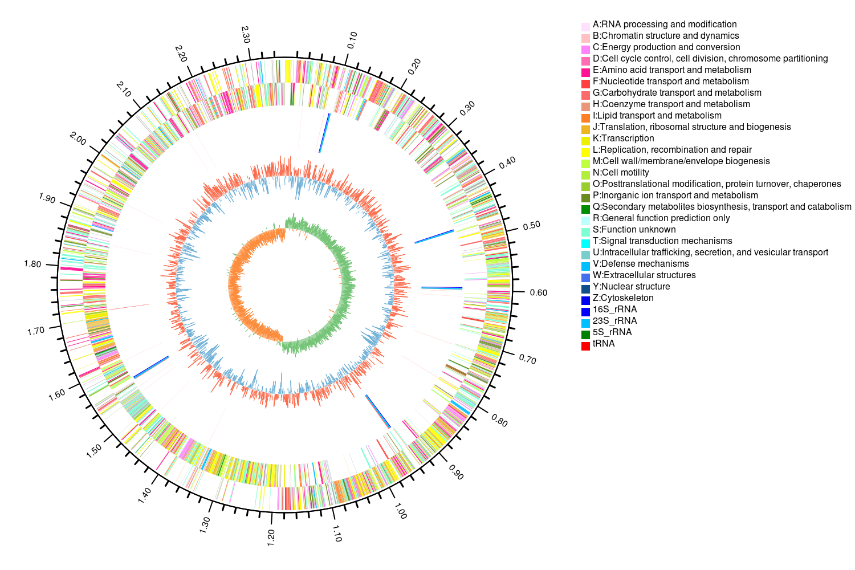


**Supplementary Fig.S2.** Genome map of *Levilactobacillus brevis* YSJ3 chromosome


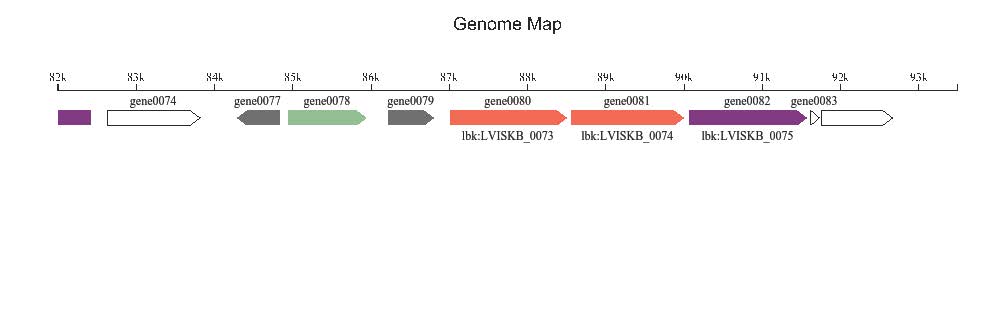


**GadB**

**GadC**

**GadR**

**Supplementary Fig.S3.** Genome map of gad operon including GadB, GadC and GadR from *Levilactobacillus brevis* YSJ3


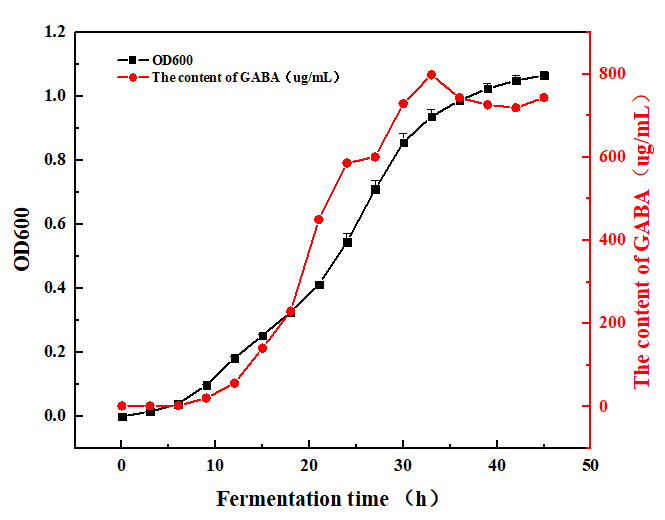


**Supplementary Fig.S4.** GABA production by *Levilactobacillus brevis* YSJ3 in vitro from MRS broth
